# Adolescent Treatment Landscape of Depression, Suicidality, and Substance Use Disorder in the US

**DOI:** 10.1001/jamahealthforum.2025.2647

**Published:** 2025-08-29

**Authors:** Dennis Lee, Stacie B. Dusetzina, Stephen W. Patrick, John A. Graves, Carrie E. Fry

**Affiliations:** 1 Department of Health Policy and Management, Fielding School of Public Health, University of California, Los Angeles; 2 Department of Health Policy, Vanderbilt University School of Medicine, Vanderbilt University, Nashville, Tennessee; 3 Department of Health Policy and Management, Schaeffer Center for Health Policy and Economics, University of Southern California, Los Angeles; 4 Vanderbilt-Ingram Cancer Center, Nashville, Tennessee; 5 Department of Health Policy and Management, Rollins School of Public Health, Emory University, Atlanta, Georgia; 6 Department of Pediatrics, Division of Neonatology, Emory School of Medicine, Atlanta, Georgia; 7 Health Services Research Center, Emory University, Atlanta, Georgia

## Abstract

**Importance:**

Recent trends in drug-related overdoses among adolescents have highlighted the need for mental health and substance use disorder (SUD) treatment. However, the extent of these treatment gaps is understudied.

**Objective:**

To characterize the factors associated with the diagnosis of and treatment for mental health and SUD for adolescents.

**Design, Setting, and Participants:**

This cross-sectional study used survey-weighted descriptive statistics and χ^2^ tests to estimate differences in characteristics and treatment receipt and included US adolescents and young adults aged 12 to 20 years who participated in the National Survey on Drug Use and Health in 2021 and 2022. Data were analyzed from February 2024 to February 2025.

**Main Outcomes and Measures:**

Primary outcomes included the prevalence of depression and suicidality-related mental health diagnoses, SUDs, and treatment rates for both conditions. Additional measures included treatment setting, socioeconomic and demographic characteristics, and health insurance–related factors.

**Results:**

From 2021 to 2022, 13% of participants had SUD and 24% had a mental health diagnosis during the previous year (mean [SD] age, 16.0 [2.5] years; 48.4% female individuals; 6.1% Asian, 13.9% Black, 25.7% Hispanic, and 49.9% White individuals). Only 10% of participants with SUD and 51% of adolescents with mental health diagnoses received treatment for their conditions, with higher rates of treatment among adolescents with comorbid SUD and mental health diagnoses. When comparing adolescents (aged 12-17 years) and young adults (aged 18-20 years) with SUD for treatment receipt, reductions were found in any mental health treatment (63% vs 51%; *P* = .03) and any SUD treatment (11% vs 8%; *P* = .01). Moreover, these lower rates were also found in more resource-intensive treatment settings, such as inpatient mental health care (14% vs 9%; *P* = .02) and specialty mental health facilities (47% vs 33%; *P* = .003). However, adolescents with opioid use disorder were less likely to receive medication treatment (11% vs 28%; *P* = .02). Treatment differences were associated with socioeconomic and insurance coverage factors. Compared with adolescents, young adults with SUD experienced increased poverty rates (20% vs 26%; *P* = .02), uninsurance rates (5% vs 10%; *P* = .05), and private insurance rates (49% vs 56%; *P* = .02) while receiving decreased Medicaid coverage (47% vs 33%; *P* < .001) and government assistance (34% vs 25%; *P* = .001).

**Conclusions and Relevance:**

The results of this cross-sectional survey study suggest that adolescents and young adults with SUDs rarely received treatment. Adolescents are especially vulnerable to treatment gaps once reaching young adulthood, and medications for opioid use disorder are systematically underused, especially for adolescents.

## Key Points

**Question:**

What is the treatment landscape for adolescents and young adults (aged 12-20 years) with depression and suicidality-related mental health and substance use disorders (SUDs) from 2021 to 2022 in the United States?

**Findings:**

In this cross-sectional survey study of 32 598 adolescents and young adults from the National Survey on Drug Use and Health, 51% of adolescents with depression and suicidality-related diagnoses and 10% of adolescents with SUD received treatment for their conditions. Those (aged 18-20 years) with SUD had significantly lower treatment rates for mental health (51% vs 63%) and SUD (8% vs 11%) compared with adolescents (aged 12-17 years).

**Meaning:**

The study results suggest that adolescents and young adults face unique systematic treatment barriers in SUD care that are exacerbated during the transition to young adulthood.

## Teaser

This cross-sectional survey study examines the factors associated with the diagnosis of and treatment for mental health and substance use disorder for US adolescents.

## Introduction

Drug-related overdoses are the third most common cause of death for US individuals younger than 20 years.^1^ Despite declining trends in overall adolescent illicit drug use,^2,3,4,5^ the relative increase in drug-related overdose deaths for high school–aged adolescents had tripled that of the general population from 2019 to 2021.^6^ Although such increases in mortality have been primarily associated with unintentional poisonings, mostly via fentanyl,^3,6,7^ 41% of these decedents had mental health histories, 35% used opioids before, and nearly one-fourth died after using counterfeit drugs.^2^ Adolescents often unwittingly purchased fentanyl-laced pills that were disguised as antianxiety medication (eg, alprazolam) or prescription opioids, such as oxycodone.^2,3,8^

Prior studies have shown that most adolescents and young adults with SUDs have psychiatric comorbidities,^9,10,11^ which have a bidirectional association with substance use.^10,11,12^ Among adolescents who died of a drug-related overdose from 2019 to 2021, 24% received mental health treatment, 19% received a diagnosis of depression, and 15% displayed suicidal ideation or self-harming behavior.^2^ Moreover, trauma and adverse childhood experiences (ACEs) are strongly associated with SUDs; 84% of adults with SUDs experienced at least 4 ACEs during their lifetimes compared with 13% in the general population.^11^ Evidence-based mental health treatments for children with ACEs or a mental health condition can reduce the risk for SUD later in life.^11^ Thus, mental health treatment may be one avenue through which the harms associated with adolescent substance use can be curbed.

Effective SUD treatments may also improve outcomes, but these are underused. Only 3% of adolescents who died of overdose were receiving SUD treatment at the time of death, and only 11% had a history of SUD treatment.^2^ In contrast, 35% had some history of opioid use, and 14% had survived an overdose before.^2^ Most barriers to SUD treatment are structural, including factors associated with access to health care clinicians and institutions, costs, and legal barriers.^13^ Because adolescents are especially vulnerable during their transition to adulthood,^14^ understanding how barriers affect adolescents (aged 12-17 years) and young adults (aged 18-20 years) differently in terms of access to mental health and SUD treatment is important.

Young adults may face greater insurance and cost-related issues, whereas adolescents may face greater legal issues to treatment. In 2016, young adults aged 19 to 25 years had greater rates of uninsurance and private health insurance, lower rates of public insurance, lower health care utilization, and higher rates of delayed care due to cost compared with adolescents aged 15 to 18 years.^15^ In contrast, although medications for opioid use disorder (MOUDs) are some of the most effective treatments for opioid use disorder,^16,17^ only buprenorphine is indicated for use in adolescents, and its use is limited to those who are at least age 16 years.^10,18,19^ Despite endorsement from the American Academy of Pediatrics,^18^ the use of MOUDs for adolescents is scarce. In the Medicaid population, less than 8% of adolescents (aged 12-17 years) with opioid use disorder (OUD) received any MOUD, which is considerably lower than the 32% of young adults receiving MOUD treatment (aged 18-25 years).^20,21^

In this study, we characterized the diagnosis and receipt of treatments for adolescents (aged 12-20 years) for mental health and SUDs using a nationally representative survey. We delineated differences in treatment use due to socioeconomic factors, demographic background, and mental health and SUD status to inform potential policy levers to address such disparities.

## Methods

### Study Population

The study population consisted of adolescents aged 12 to 20 years who were included in the 2021 and 2022 National Survey on Drug Use and Health (NSDUH). The NSDUH is an annual, nationally representative household survey of the US noninstitutionalized civilian population.^22,23^ The NSDUH produces prevalence estimates of substance use, mental health diagnoses, and associated treatments.^22,23^ The survey uses a complex stratified sampling design and explores age-specific topics for adolescents and young adults.^22,23^ The Vanderbilt University institutional review board determined that this study was not human subjects research, and informed consent was waived due to the use of deidentified data. In addition, we followed the Strengthening the Reporting of Observational Studies in Epidemiology (STROBE) reporting guideline for cross-sectional studies.

### Variables of Interest

For mental health diagnoses, we measured experiencing a major depressive episode (MDE), more severe depression (ie, MDE to the point of role impairment), and suicidal ideation during the previous year. We noted the inclusion of any of these variables as having a mental health diagnosis. The NSDUH used diagnostic criteria from *Diagnostic and Statistical Manual of Mental Disorders* (Fifth Edition (*DSM-5*) to screen respondents for MDEs.^22,23^

To classify substance use, we included the presence of any SUD and OUD as defined by *DSM-5* diagnostic criteria in the NSDUH. In addition, we included measures of any opioid use and opioid misuse (defined as taking medications in any undirected manner, including using unprescribed medications, or using medications in greater amounts or for longer periods than prescribed) during the previous year.^22,23^

We measured the receipt of any mental health treatment as having received telehealth, prescriptions, outpatient, or inpatient treatment for mental health during the previous year. Additionally, we included the receipt of treatment through inpatient care, telehealth, school resources, or a specialty facility. For adolescents who had a mental health diagnosis during the previous year, we captured receipt of counseling from a health care professional (including physicians, psychologists, social workers, and therapists), prescription medications, and alternative mental health treatment from nonmedical sources (eg, religious leaders and herbalists).

For SUD treatments, we measured the receipt of health care for alcohol, drug, and SUDs through inpatient, outpatient, telehealth, and specialty facilities care. We defined the receipt of MOUDs by combining the receipt of any MOUD and the receipt of methadone or buprenorphine if the respondent screened positive for OUD. See eTable 1 in Supplement 1 for details on measure construction.

### Covariates

We included demographic, socioeconomic, and mental health–related variables as covariates: self-reported sex (female or male); racial and ethnic identity (Asian, Black, Hispanic, non-Hispanic White, and other [including non-Hispanic multiracial, non-Hispanic Native American/Alaska Native, and non-Hispanic Native Hawaiian/other Pacific Islander]); age group (12-13, 14-15, 16-17, and 18-20 years); health insurance status (uninsured, Medicaid, private, or other); receipt of government assistance (including Supplemental Security Income and Supplemental Nutrition Assistance Program); experience of poverty; or arrest history.

### Analyses

We estimated the survey-weighted prevalence of mental health diagnoses, SUDs, and receipt of treatment for both conditions. We also estimated the survey-weighted prevalence of SUD by respondents’ sociodemographic characteristics and the presence of a comorbid mental health condition. We used the Pearson χ^2^ test with the Rao and Scott adjustment for complex surveys to estimate differences in characteristics and treatment receipt by the presence of SUD.^24,25^ All statistical tests used an α level of .05.

We also estimated the rate of mental health treatment use by SUD status for those with a mental health diagnosis, rates of formal and informal mental health treatment and SUD treatment by age for those with SUD, and receipt of MOUD for those with OUD. Finally, we compared demographic, socioeconomic, mental health, and opioid use variables by treatment status for those with SUD and by mental health diagnoses, respectively.

### Sensitivity Analyses

Because our main analyses involved bivariate comparisons, we included multivariable modified Poisson regressions as sensitivity analyses to calculate adjusted estimates. We included regressions when the outcomes of interest were substance use disorder (eTable 6 in Supplement 1), an indicator for young adult status (eTable 7 in Supplement 1), and treatment receipt for SUD and mental health (eTable 8 in Supplement 1). Covariates for eTables 6 and 8 in Supplement 1 included demographic (eg, sex, race and ethnicity, and age) and socioeconomic variables (eg, insurance type, receipt of government aid, poverty status, and arrest history), any mental health diagnosis, and opioid use when appropriate. Covariates for eTable 7 in Supplement 1 included the receipt of mental health treatment, substance use treatment, and socioeconomic variables. All covariates from the regressions were included in the supplemental tables. Although there were slight variations, the significant associations from our bivariate analyses were generally consistent with those from our multivariable regressions.

## Results

Our sample included 32 598 adolescents, who represented a weighted population of 38 782 587 from ages 12 to 20 years in the US. Of our sample, 13% met the diagnostic criteria for SUD (Table 1). Those with SUD were more likely to be female (53% vs 48%; *P* < .001); identify as Hispanic, non-Hispanic White, or another racial identity (27% vs 25% for Hispanic, 53% vs 49% for non-Hispanic White, and 6% vs 4% for other; *P* < .001), and older (age 16-20 years, 79% vs 51%; *P* < .001) than those without an SUD.

**Table 1.** Demographic Characteristics for Adolescents With and Without Substance Use Disorder (SUD) in the US From 2021 to 2022

Characteristic	%			*P* value
	Overall (N = 32 598; weighted N = 38 782 587)	SUD (n = 4082; weighted n = 5 081 759)	No SUD (n = 28 516; weighted n = 33 700 828)	
Total	100	13.10	86.90	
Sex				
Female	48.39	53.46	47.63	<.001
Male	51.61	46.54	52.37	
Race and ethnicity				
Asian	6.07	2.96	6.54	
Black	13.86	11.29	14.25	<.001
Hispanic	25.69	27.02	25.49	
White	49.90	52.82	49.46	
Other^a^	4.47	5.91	4.26	
Age group, y				
12-13	22.35	6.24	24.77	<.001
14-15	22.61	14.81	23.79	
16-17	21.76	25.39	21.21	
18-20	33.29	53.56	30.23	
**Socioeconomic factors** ^b^				
Insurance status				
None	6.78	7.85	6.61	.13
Medicaid	39.30	39.34	39.30	.98
Private	53.30	52.59	53.41	.60
Other	6.38	5.87	6.46	.38
Government assistance	26.96	28.88	26.67	.10
Poverty	22.71	23.25	22.62	.59
Arrested	2.45	8.41	1.56	<.001
Mental health				
Any diagnosis	24.50	46.29	21.21	<.001
MDE	19.86	36.62	17.31	<.001
MDE with role impairment	14.62	29.27	12.42	<.001
Suicidal ideation	15.31	33.96	12.48	<.001
Opioid use				
Any opioid use	14.35	28.89	12.16	<.001
Opioid misuse	2.19	9.64	1.07	<.001
Opioid use disorder	1.07	8.20	0.00	<.001

Abbreviation: MDE, major depressive episode.

^a^ Included non-Hispanic multiracial, non-Hispanic Native American/Alaska Native, and non-Hispanic Native Hawaiian/other Pacific Islander individuals.

^b^ All socioeconomic, mental health, and substance use measures pertain for the previous year.

Among respondents, those who met the diagnostic criteria for SUDs had also experienced past-year mental health diagnoses at higher rates (46% vs 21%; *P* < .001) than those who did not. Specifically, respondents with SUD were more likely to experience a severe depressive episode (29% vs 12%; *P* < .001) and suicidal ideation (34% vs 12%; *P* < .001) than respondents without SUD. Additionally, respondents with SUD were more likely to have used (29% vs 12%; *P* < .001) or misused (10% vs 1%; *P* < .001) opioids during the previous year. Among those with SUD, 8% met the criteria for OUD.

We found no statistically significant differences in health insurance status or composition, receipt of government assistance, or poverty by SUD. However, we found a significant difference in the arrest history (8% vs 2%; *P* < .001) and stark differences in socioeconomic factors among those with SUD by age. Compared with adolescents (age 12-17 years), young adults (age 18-20 years) with SUD had lower receipt of government assistance (34% vs 25%; *P* = .001) and a higher likelihood of experiencing poverty (20% vs 26%; *P* = .02) or having been arrested (6% vs 10%; *P* = .03). Although young adults had a small (5%) relative decrease in health insurance coverage compared with adolescents (95% vs 90%; *P* = .05), they were much less likely to have Medicaid coverage (47% vs 33%; *P* < .001) and more likely to have private insurance (49% vs 56%; *P* = .02).

### Overall Treatment Receipt

Among all adolescents with a mental health diagnosis during the previous year, rates of treatment differed by SUD status (Table 2). Those with SUD were more likely to receive any mental health treatment compared with those without SUD (57% vs 49%; *P* = .01); this equated to a treatment gap of 550 520 adolescents with a mental health diagnosis without treatment. Specifically, those with SUD were more likely to receive professional help (49% vs 40%; *P* = .001), prescriptions to treat depression (37% vs 23%; *P* < .001), inpatient mental health care (12% vs 5%; *P* < .001), virtual health (39% vs 29%; *P* < .001), and treatment through a specialty facility (40% vs 33%; *P* = .002) (Table 3). However, there were no statistically significant differences in terms of mental health care received through school or alternative mental health treatment.

**Table 2.** Mental Health Treatment by Substance Use Disorder (SUD) Status for Those With a Mental Health Diagnosis

Treatment^a^	%			*P* value
	Overall	SUD (n = 1907; weighted n = 2 315 614)	No SUD (n = 5805; weighted n = 7 045 605)	
Percentage	100	24.74	75.26	
Any mental health treatment	51.36	57.25	49.43	.01
Professional counseling^b^^,^^c^	42.07	49.25	39.73	.001
Medications^b^	25.96	36.54	22.54	<.001
Counseling or medications^b^	45.11	54.92	41.93	<.001
Alternative help^b^^,^^d^	3.22	3.50	3.13	.62
Inpatient	6.35	11.57	4.65	<.001
Virtual treatment	31.34	38.89	28.87	<.001
School resources	19.13	19.00	19.18	.93
Specialty facility	34.81	40.33	33.00	.002

^a^ The denominator includes respondents who had experienced a mental health diagnosis (a major depressive episode or suicidal ideation) during previous year.

^b^ Questions were asked to adolescents who experienced negative feelings.

^c^ Professional counseling from a health care professional included medical professionals, such as physicians, psychiatrists, nurses, and occupational therapists, and mental health professionals, including psychologists, psychotherapists, social workers, counselors.

^d^ Alternative help included counseling from a religious leader or advisor and providers of alternative medicine, such as herbalists, acupuncturists, or message therapists.

**Table 3.** Differences in Socioeconomic Status and Treatment Receipt by Age for Adolescents With Substance Use Disorder (SUD) From 2021 to 2022

Category	Variable	Age, %		*P* value
		12-17 y (n = 1981; weighted n = 2 360 211)	18-20 y (n = 2101; weighted n = 2 721 549)	
Socioeconomic variables	Uninsured	5.43	9.94	.05
	Medicaid	47.01	32.69	<.001
	Private insurance	49.08	55.65	.02
	Other insurance	4.80	6.80	.07
	Government assistance	33.64	24.75	.001
	Poverty	20.13	25.95	.02
	Arrested	6.42	10.15	.03
Mental health treatment^a^	Any mental health treatment	62.75	51.37	.03
	Professional counseling	49.84	48.58	.80
	Medications	37.14	35.84	.80
	Professional counseling or medications	53.94	56.06	.67
	Alternative help	3.86	3.08	.68
	Inpatient	14.28	8.67	.02
	Telehealth	38.64	39.15	.91
	School resources	29.89	7.40	<.001
	Specialty facility	47.15	33.06	.003
Substance use treatment	Any SUD treatment	11.45	8.03	.01
	Help for alcohol	3.10	2.26	.33
	Help for drugs	7.89	5.00	.03
	Inpatient	3.03	2.16	.30
	Outpatient	3.55	3.21	.72
	Telehealth	3.78	2.79	.26
	Specialty facility	5.76	3.95	.06

^a^ The denominator for the mental health treatment variables is adolescents with SUD who had a mental health diagnosis during the previous year.

For adolescents with SUDs, we found no differences in SUD treatment receipt by sex, race, overall and private health insurance coverage, poverty, or suicidal ideation (Table 4). However, SUD treatment receipt was higher for those with Medicaid (11% vs 9%; *P* = .04) and other health insurance (18% vs 9%; *P* = .01), adolescents (55% vs 46%; *P* = .012), those with a history of arrest (19% vs 9%; *P* < .001), those who had received government assistance (13% vs 8%; *P* = .04), those with MDE (12% vs 8%; *P* = .003) and MDE to the point of role impairment (13% vs 8%; *P* = .001), and any opioid use (15% vs 7%; *P* < .001) and misuse (25% vs 8%; *P* < .001).

**Table 4.** Treatment Receipt by Need and Adolescent Characteristics

Characteristic	SUD treatment for SUD (n = 4082; weighted n = 5 081 759)			MH treatment for MH diagnosis (n = 7656; weighted n = 9 308 350)^a^			
	%		*P* value	%		*P* value	
	Row = 1	Row = 0		Row = 1	Row = 0		
Demographic							
Female	10.90	8.15	.12	55.53	43.45	<.001	
Male	8.15	10.90		43.45	55.53		
Asian	14.80	9.46	.64	47.39	51.58	<.001	
Black	9.02	9.69		36.29	53.31		
Hispanic	8.68	9.96		45.96	53.17		
White	10.12	9.06		57.91	44.03		
Other^b^	7.97	9.72		48.23	51.54		
Age group, y							
12-13	15.66	9.21	.02	50.43	51.53	.002	
14-15	12.66	9.09		57.61	49.46		
16-17	9.71	9.58		51.95	51.15		
18-20	8.03	11.45		47.13	53.62		
Socioeconomic							
Insured	9.89	6.37	.26	52.29	36.35	.001	
Medicaid	11.41	8.45	.04	51.30	51.40	.96	
Private insurance	8.63	10.71	.15	53.84	48.23	.002	
Other insurance	18.21	9.08	.01	46.47	51.72	.14	
Government assistance	12.56	8.42	.04	52.46	50.97	.52	
Poverty	10.32	9.41	.60	48.68	52.03	.19	
Arrested	18.91	8.81	<.001	58.04	51.22	.23	
MH type, severity							
Any MH condition	11.34	8.25	.04	NA	NA		NA
MDE	11.97	8.23	.003	53.17	43.39	.001	
MDE role impairment	12.78	8.25	.001	58.33	40.90	<.001	
Suicidal ideation	10.86	8.36	.08	54.78	45.41	.002	
SUD							
Any SUD	NA			57.25	49.43	0.01	
Any opioid use	15.12	7.38	<.001	56.69	50.00	.02	
Opioid misuse	25.31	7.94	<.001	62.39	50.83	.01	
Treatment rates overall	9.62	90.38	NA	51.36	48.64	NA	

Abbreviations: MDE, major depressive episode; MH, mental health; NA, not applicable; SUD, substance use disorder.

^a^ Mental health diagnoses included experiencing an MDE or suicidal ideation during the previous year.

^b^ Included non-Hispanic multiracial, non-Hispanic Native American/Alaska Native, and non-Hispanic Native Hawaiian/other Pacific Islander individuals.

For those with mental health diagnoses, mental health treatment receipt was higher for female individuals (56% vs 43%; *P* < .001), non-Hispanic white adolescents (58% vs 44%; *P* < .001), those with any health insurance (52% vs 36%; *P* = .001), those with more severe depression (58% vs 41%; *P* < .001), suicidal ideation (55% vs 45%; *P* = .002), a co-occurring SUD (57% vs 49%; *P* = .01), and opioid misuse (62% vs 51%; *P* = .01). Mental health treatment receipt was lower for young adults (47% vs 54%; *P* = .002) and Hispanic (46% vs 53%) or non-Hispanic Black adolescents (36% vs 53%; *P* < .001). We found no differences by other types of health insurance (including Medicaid), receipt of government assistance, poverty status, or arrests.

### Treatment Receipt by Age

When comparing treatment receipt between adolescents (aged 12-17 years) and young adults (aged 18-20 years) with SUDs, we found lower receipt of any mental health treatment for those with a mental health condition (63% vs 51%; *P* = .03) and in the receipt of treatment for SUDs (11% vs 8%; *P* = .01). This accounted for 127 332 and 93 152 young adults not receiving mental health and SUD treatment, respectively. We found no statistically significant differences in the receipt of mental health treatments through a health care professional, prescriptions, telehealth, or alternative treatments by age; however, we found that, compared with adolescents (aged 12-17 years), young adults (aged 18-20 years) were less likely to receive inpatient care (14% vs 9%; *P* = .02), mental health treatment through specialty facilities (47% vs 33%; *P* = .003), and treatment through school (30% vs 7%; *P* < .001).

In terms of SUD, we found that treatments for alcohol use disorder and care provided in inpatient, outpatient, telehealth, and specialty facilities did not vary by age. However, treatment rates for illicit drug use were lower (8% vs 5%; *P* = .03) for young adults compared with adolescents. Finally, we found that receipt of any MOUD (11% vs 28%; *P* = .02) and buprenorphine specifically (6% vs 25%; *P* = .003) was higher among young adults than adolescents with OUD. There was no difference in the receipt of methadone by age (Table 5).

**Table 5.** Receipt of Medications for Opioid Use Disorder (MOUD) for Those With Opioid Use Disorder During the Previous Year

MOUD	Age, y	%	Weighted No.	No.	*P* value
Any MOUD	12-17	11.12	26 702	34	.02
	18-20	28.13	42 323	24	
Methadone	12-17	2.99	7196	8	.70
	18-20	3.86	5806	6	
Buprenorphine	12-17	6.09	14 901	22	.003
	18-20	25.01	37 627	18	

## Discussion

In this cross-sectional survey study, we found significant treatment gaps among adolescents, with only 51% with a mental health diagnosis and 10% with a SUD having received any treatment for the respective conditions. Although the prevalence of mental health diagnoses was considerably higher among adolescents with SUD compared with those without, the presence of co-occurring SUD was associated with an increased likelihood of receiving mental health treatment, including counseling and medication for a depressive episode. This may reflect either greater need or willingness to treat psychiatric conditions for these adolescents, particularly with medications.

Adolescents with SUD older than 18 years faced even larger treatment gaps. We found significant dropoffs between adolescents and young adults for any mental health and SUD treatment. Moreover, these drops were found in resource-intensive treatment settings, such as inpatient mental health care and mental health specialty facilities. We observed a doubling in the likelihood of having no insurance, 13% increase in private insurance, and 30% reduction in Medicaid coverage for adolescents aged 12 to 17 years vs those aged 18 to 20 years. Patients with Medicaid have significantly lower cost-sharing and higher utilization compared with patients with private insurance or no insurance.^26,27,28^ For example, the monthly mean out-of-pocket costs for buprenorphine were $2.10 with Medicaid, $91.50 with commercial insurance, and $249.90 with self-pay.^29^ Because individuals aged 18 to 20 years are more likely to have private insurance or be uninsured and experience poverty compared with adolescents, young adults with SUD are likely particularly vulnerable to these out-of-pocket costs. This is especially salient for resource-intensive treatment settings, such as residential treatment, where the median upfront cash cost for adolescents may exceed $18 000.^30^

Even with insurance coverage, administrative and payment issues present substantial barriers to behavioral health treatment.^31,32^ Psychiatrists and buprenorphine-providing physicians often have small or solo practices,^31,33,34^ where the relatively low reimbursements and high administrative costs make accepting insurance less appealing.^31,33^ Only 72% (52% for Medicaid) of buprenorphine-prescribing physicians and 59% (39% for Medicaid) of psychiatrists accepted private insurance.^34,35^ Secret shopper studies have shown that obtaining an appointment with buprenorphine-prescribing providers with Medicaid is more difficult than with private insurance or no insurance.^36,37^

Although 98% of pediatricians accept private insurance and 85% accept Medicaid,^38^ adolescents with SUD may still face challenges receiving treatment. A recent survey found that although almost all (92%) pediatricians viewed screening for SUD as their responsibility, only 20% felt that treating SUD was their responsibility.^39^ OUD seemed particularly challenging for pediatricians; only 48% of pediatricians felt prepared to counsel adolescents on opioid use compared with 87% on alcohol use and 81% for cannabis.^39^ Further, while 24% of surveyed pediatricians had given an adolescent an OUD diagnosis, only 6% ever prescribed an MOUD. Less than 2% of all buprenorphine prescriptions are written by pediatricians.^39,40^

Adolescent access to specialty SUD treatment facilities is also worrisome; only 26% of addiction treatment facilities and less than half of residential addiction treatment facilities treat adolescents.^30,41^ Additionally, of these adolescent-serving facilities, less than a third provide buprenorphine.^42^ These factors contribute to the low overall MOUD treatment rates among adolescents. We found that adolescents were much less likely to receive any MOUD and buprenorphine specifically. Improving payment rates for behavioral health care professionals is one step to increasing access, as is increasing training around SUD identification and treatment during medical school and residency for pediatricians.^43^

### Limitations

This study had limitations. First, our analyses were descriptive; causal relationships should not be implied. Second, the NSDUH is a household survey of noninstitutionalized civilians, which excludes unhoused or unstably housed, incarcerated, or institutionalized adolescents who are receiving inpatient or residential treatment. This group may be particularly important given that these youth are significantly more likely to have serious mental health diagnoses and substance use.^44,45^ Third, NSDUH lacks questions on non–depression-related mental health conditions, such as anxiety disorder, attention-deficit/hyperactivity disorder, bipolar disorder, or ACEs, that may provide greater insight to the psychiatric need and relationships with SUDs in adolescents. Fourth, our age categories were limited by the groupings available. We could not disaggregate the category for individuals aged 18 to 20 years to better capture dropoffs in Medicaid enrollment at age 19 years. Fifth, the NSDUH did not ask which MOUDs were used. To better capture the receipt of MOUDs, we included the use of buprenorphine or methadone as directed by a clinician for those with OUD. However, this may have resulted in including adolescents with OUD who were being treated for pain.

## Conclusions

The results of this cross-sectional survey study suggest that many adolescents have an SUD and nearly a quarter have a depression or suicidality-related mental health diagnosis. However, only one-tenth of adolescents with SUD and roughly half of adolescents with mental health diagnoses received treatment, although those with a mental health diagnosis and SUD had higher treatment rates. We also found treatment gaps for young adults for mental health and SUD that were likely associated with socioeconomic vulnerability, differences in insurance coverage, and issues associated with reimbursement. Given the unique barriers that adolescents face, policies to improve pediatrician training, insurance coverage and out-of-pocket costs, clinician reimbursement, and access to evidence-based medications are greatly needed.

## Notes

Supplement 1. eTable 1. Variable Construction from the National Survey on Drug Use and Health eTable 2. Demographic Characteristics for Adolescents with and without Substance Use Disorder in the United States from 2021-2022 eTable 3. Mental Health Treatment by Substance Use Disorder Status for those with a Mental Health Diagnosis eTable 4. Differences in socioeconomic status and treatment receipt by age for adolescents with substance use disorder, 2021-2022 eTable 5. Treatment Receipt by Need and Adolescent Characteristics eTable 6. Combined Modified Poisson Regression for Substance Use Disorder and Demographic Characteristics eTable 7. Modified Poisson Regression of Treatment Rates by Age for Adolescents with Substance Use Disorder eTable 8. SUD Combined Modified Poisson Regression for Treatment Receipt by Need and Adolescent Characteristics eReferences.

Supplement 2. Data sharing statement
