# Supplementary material for: Adolescent Treatment Landscape of Depression, Suicidality, and Substance Use Disorder in the US
Source: JAMA Health Forum. 2025 Aug 29;6(8):e252647. doi: 10.1001/jamahealthforum.2025.2647 (PMC12397884; doi:10.1001/jamahealthforum.2025.2647)
Supplement: Supplement 1. — eTable 1. Variable Construction from the National Survey on Drug Use and Health eTable 2. Demographic Characteristics for Adolescents with and without Substance Use Disorder in the United States from 2021-2022 eTable 3. Mental Health Treatment by Substance Use Disorder Status for those with a Mental Health Diagnosis eTable 4. Differences in socioeconomic status and treatment receipt by age for adolescents with substance use disorder, 2021-2022 eTable 5. Treatment Receipt by Need and Adolescent Characteristics eTable 6. Combined Modified Poisson Regression for Substance Use Disorder and Demographic Characteristics eTable 7. Modified Poisson Regression of Treatment Rates by Age for Adolescents with Substance Use Disorder eTable 8. SUD Combined Modified Poisson Regression for Treatment Receipt by Need and Adolescent Characteristics eReferences. [file jamahealthforum-e252647-s001.pdf]

Supplemental Online Content

Lee D, Dusetzina SB, Patrick SW, Graves JA, Fry CE. Adolescent treatment landscape of depression, suicidality, and substance use disorder in the US. *JAMA Health Forum*. 2025;6(8): e252647. doi:10.1001/jamahealthforum.2025.2647

eTable 1. Variable Construction from the National Survey on Drug Use and Health
eTable 2. Demographic Characteristics for Adolescents with and without Substance Use Disorder in the United States from 2021-2022
eTable 3. Mental Health Treatment by Substance Use Disorder Status for those with a Mental Health Diagnosis
eTable 4. Differences in socioeconomic status and treatment receipt by age for adolescents with substance use disorder, 2021-2022
eTable 5. Treatment Receipt by Need and Adolescent Characteristics
eTable 6. Combined Modified Poisson Regression for Substance Use Disorder and Demographic Characteristics
eTable 7. Modified Poisson Regression of Treatment Rates by Age for Adolescents with Substance Use Disorder
eTable 8. SUD Combined Modified Poisson Regression for Treatment Receipt by Need and Adolescent Characteristics
eReferences.

This supplemental material has been provided by the authors to give readers additional information about their work.

© 2025 Lee D et al. *JAMA Health Forum*.

I) Variable Construction

Appendix Table 1 includes the variables used to construct our measures from the National Survey on Drug Use and Health (NSDUH).[1,2] Our general approach was to select the variables that retained data consistency between the 2021 and 2022 versions as inclusively as possible. We chose the imputed recode versions for every variable wherever possible as recommended and recoded missing values in a manner consistent with the imputed recode when necessary. Please see the codebooks for exact definitions.[1,2]

<table>
<tr><th colspan="4">eTable 1: Variable Construction from the National Survey on Drug Use and Health</th></tr>
<tr><th>Variable Class</th><th>Variables</th><th>NSDUH[a] Variables (2021)</th><th>NSDUH Variables (2022)</th></tr>
<tr><td rowspan="3">Demographics</td><td>Female</td><td colspan="2">IRSEX</td></tr>
<tr><td>Race</td><td colspan="2">NEWRACE2[b]</td></tr>
<tr><td>Age</td><td colspan="2">CATAG7</td></tr>
<tr><td rowspan="7">Socio-economic</td><td>Insured</td><td colspan="2">IRINSUR4</td></tr>
<tr><td>Insurance: Medicaid</td><td colspan="2">IRMCDCHP</td></tr>
<tr><td>Insurance: private</td><td colspan="2">IRPRVHLT</td></tr>
<tr><td>Insurance: other</td><td colspan="2">OTHINS[c]</td></tr>
<tr><td>Received Government Assistance</td><td colspan="2">GOVTPROG[d]</td></tr>
<tr><td>Poverty</td><td colspan="2">POVERTY3</td></tr>
<tr><td>Arrested</td><td colspan="2">BOOKED</td></tr>
<tr><td rowspan="4">Mental Health</td><td>Major Depressive Episode in the Past Year</td><td colspan="2">AMDEYR, YMDEYR</td></tr>
<tr><td>Major Depressive Episode with Role Impairment</td><td colspan="2">MDEIMPY</td></tr>
<tr><td>Suicidal Ideation</td><td colspan="2">YUSUITHKYR</td></tr>
<tr><td>Any Mental Health Issue[e]</td><td colspan="2">AMDEYR, YMDEYR, YUSUITHKYR</td></tr>
<tr><td rowspan="4">Substance Use</td><td>Any Opioid Use in the Past Year</td><td colspan="2">OPIANYYR</td></tr>
<tr><td>Opioid Misuse in the Past Year</td><td colspan="2">OPINMYR</td></tr>
<tr><td>Opioid Use Disorder</td><td colspan="2">UD5OPIANY</td></tr>
<tr><td>Substance Use Disorder</td><td colspan="2">UD5ILALANY</td></tr>
<tr><td></td><td>Received Help from a Healthcare Professional[f,g]</td><td colspan="2">YHLTMDE, AHLTMDE</td></tr>
</table>

© 2025 Lee D et al. *JAMA Health Forum*.

**Mental Health Treatments**	Received Prescriptions[f]	YRXMDEYR, ARXMDEYR	
	Received Help from a Healthcare Professional or Prescriptions[f]	YMDEHPRX, AHLTMDE, ARXMDEYR	
	Alternative Help[h]	YALTMDE, AALTMDE	
	Any Mental Health Service[h]	ANYMHOVR, AMHTXRC4	MHTRTPY
	Inpatient Mental Health Treatment	AMHINP2, ANYMHIN2	MHTINPPY
	Through School	ANYMHED2, MHLSCHL4	IRMHTOUTSCHL
**Variable Class**	**Variables**	**NSDUH[a] Variables (2021)**	**NSDUH Variables (2022)**
**Mental Health Treatments**	Any Specialty Facility Treatment	ANYSMH2, AMHINP2, MHLMNT4, MHLDTMT4, MHLTHER4	IRMHTINHOSP, IRMHTINRSMH, IRMHTINRHAB, IRMHTOUTMHCR, IRMHTOUTRHAB, IRMHTOUTTHRP
	Talked with Parents about Substance Use	PRTALK3	
**Substance Use Treatments**	Received Help for Alcohol	TXYRALC	SUTRTALCPY
	Received Help for Drugs	TXYRILL	SUTRTDRGPY
	Specialty Inpatient[i]	TXYRHOSIL, TXYRRESIL	IRSUTINHOSP, IRSUTINRHAB
	Specialty Outpatient[j]	TXYROUTPT2, TXYRDRPRV2	IRSUTOUTDOC, IRSUTOUTRHAB
	Telehealth	TXYRTELE2	IRSUTPHVID
	Any Medications for Opioid Use Disorder[k]	OPMATYR2	IRSUTRXDRG
	Methadone	RXMTDNANY	SUTRTPY
	Buprenorphine	RXBUPRANY	

[a] National Survey on Drug Use and Health
[b] Race categories were partitioned into the following categories: "Hispanic", "Non-Hispanic White", "Non-Hispanic Black", "Non-Hispanic Asian", and "Non-Hispanic Other". The "Non-Hispanic Other" categories include individuals of Non-Hispanic Native American, Pacific Islander, and multiethnic backgrounds.
[c] Includes Medicare, military health insurance (CHAMPUS/TRICARE/VA), or some alternative form of health insurance
[d] Government assistance includes Supplemental Security Income, Supplemental Nutrition Assistance Program/Food Stamps, cash assistance, and other non-cash assistance such as help with job placements and childcare

© 2025 Lee D et al. *JAMA Health Forum*.

[e] Any mental health issue is the union of the positive values for having experienced a major depressive episode and suicidal ideation.
[f] Note: The denominator for these variables in our analysis was the presence of a mental health issue (major depressive episode or suicidal ideation) in the past year. These questions were asked to respondents experiencing negative feelings.
[g] Professional counseling from a healthcare professional includes medical professionals such as physicians, psychiatrists, nurses, and occupational therapists, and includes mental health professionals, including psychologists, psychotherapists, social workers, counselors
[h] Alternative help includes counseling from a religious leader or advisor and providers of alternative medicine such as herbalists, acupuncturists, or message therapists
[h] The 2021 and 2022 versions of the NSDUH varied in their questions regarding locations for mental health. Namely, in the 2021 version, the questions only captured inpatient mental health treatment at hospitals and residential facilities for minors (under age 18) and "overnight or longer in a hospital or other facility" for adults (over age 18). Whereas, in the 2022 version, they had captured inpatient mental health treatment at hospitals, residential substance use treatment facilities, residential mental health facilities and other facilities. Note that this variable includes outpatient mental health, but we do not include this variable separately because the outpatient variables asked for minors (under age 18) were for specialty facilities and the other outpatient locations were not consistent with that of the 2021 adults' mental health treatment section or 2022 NSDUH's sections.
[i] Though the 2022 NSDUH version measures inpatient care for substance use treatment at a residential mental health facility, we have excluded this because the 2021 NSDUH does not differentiate between inpatient or outpatient substance use treatment from residential mental health facilities. Thus, this variable only includes inpatient substance use treatment at hospitals or residential substance use rehabilitation or treatment centers.
[j] The 2022 NSDUH version measures additional outpatient substance use treatment locations that were not included in 2021, such in a therapist's office, mental health facility, and at school. We have only included measures that were included in both versions, which are through a doctor's visit or outpatient visits to substance use rehabilitation or treatment centers.
[k] We have modified the variable for the receipt of medications for opioid use disorder (MOUD) to include the both the use of prescription medications to treat opioid use and receipt of methadone (RXMTDNANY) or buprenorphine (RXBUPRANY) conditional on opioid use disorder (UD5OPIANY). This was done to increase the sensitivity of our measure, especially considering the small sample size of these variables

© 2025 Lee D et al. *JAMA Health Forum*.

II) Extended Versions of Main Tables

The following tables (eTables 2-5) are extensions of Tables 1-4 in the main draft. These include the values for the corresponding sample size (raw N) and survey-weighted populations.

eTable 2: Demographic Characteristics for Adolescents with and without Substance Use Disorder in the United States from 2021-2022										
	Overall			With Substance Use Disorder			No Substance Use Disorder			
	%	Survey-Weighted N	Raw N	%	Survey-Weighted N	Raw N	%	Survey-Weighted N	Raw N	P-value
**Overall**										
**Total**	--	38,782,587	32,598	13.10	5,081,759	4,082	86.90	33,700,828	28,516	
**Demographic**										
**Female**	48.39	18,768,618	16,107	53.46	2,716,589	2,249	47.63	16,052,029	13,858	<0.001
**Hispanic**	25.69	9,963,872	7,832	27.02	1,373,176	1,043	25.49	8,590,695	6,789	<0.001
**White**	49.90	19,352,734	15,991	52.82	2,684,043	2,027	49.46	16,668,691	13,964	
**Black**	13.86	5,375,337	4,550	11.29	573,662	501	14.25	4,801,675	4,049	
**Asian**	6.07	2,355,424	1,604	2.96	150,621	101	6.54	2,204,804	1,503	
**Other**	4.47	1,735,220	2,621	5.91	300,258	410	4.26	1,434,963	2,211	
**Aged 12-13**	22.35	8,666,002	7,580	6.24	317,213	268	24.77	8,348,789	7,312	<0.001
**Aged 14-15**	22.61	8,769,292	7,863	14.81	752,741	629	23.79	8,016,551	7,234	
**Aged 16-17**	21.76	8,437,231	7,269	25.39	1,290,258	1,084	21.21	7,146,974	6,185	
**Aged 18-20**	33.29	12,910,063	9,886	53.56	2,721,549	2,101	30.23	10,188,515	7,785	
**Socioeconomic**										
**Insured**	93.22	36,155,001	30,690	92.15	4,683,013	3,782	93.39	31,471,988	26,908	0.131
**Medicaid Insurance**	39.30	15,242,760	12,988	39.34	1,999,256	1,700	39.30	13,243,504	11,288	0.976
**Private Insurance**	53.30	20,672,334	17,370	52.59	2,672,716	2,027	53.41	17,999,618	15,343	0.595
**Other Insurance**	6.38	2,475,750	2,374	5.87	298,221	315	6.46	2,177,528	2,059	0.375
**Govt. Assistance**	26.96	10,455,743	9,002	28.88	1,467,625	1,244	26.67	8,988,118	7,758	0.104

© 2025 Lee D et al. *JAMA Health Forum*.

**Poverty**	22.71	8,793,107	7,495	23.25	1,180,424	1,041	22.62	7,612,684	6,454	0.589
**Arrested**	2.45	946,057	853	8.41	421,233	354	1.56	524,824	499	<0.001

	**Overall**			**With Substance Use Disorder**			**No Substance Use Disorder**			
	**%**	**Survey-Weighted N**	**Raw N**	**%**	**Survey-Weighted N**	**Raw N**	**%**	**Survey-Weighted N**	**Raw N**	**P-value**
**Mental Health**										
**Any Diagnosis**	24.50	9,361,219	7,712	46.29	2,315,614	1,907	21.21	7,045,605	5,805	<0.001
**MDE[a]**	19.86	7,500,163	6,171	36.62	1,824,904	1,501	17.31	5,675,259	4,670	<0.001
**MDE[a] w/ Role Impairment**	14.62	5,461,881	4,550	29.27	1,425,878	1,199	12.42	4,036,003	3,351	<0.001
**Suicidal Ideation**	15.31	5,285,790	4,335	33.96	1,548,077	1,295	12.48	3,737,713	3,040	<0.001
**Substance Use**										
**Any Opioid Use**	14.35	5,565,851	4,781	28.89	1,468,374	1,283	12.16	4,097,477	3,498	<0.001
**Opioid Misuse**	2.19	850,073	737	9.64	489,788	439	1.07	360,286	298	<0.001
**Opioid Use Disorder**	1.07	416,681	417	8.20	416,681	417	--	--	--	--

[a] MDE = Major depressive episode
All socioeconomic, mental health, and substance use measures pertain for the past year.

© 2025 Lee D et al. *JAMA Health Forum*.

eTable 3: Mental Health Treatment by Substance Use Disorder Status for those with a Mental Health Diagnosis										
	Overall			With Substance Use Disorder			Without Substance Use Disorder			
	%	Survey-Weighted N	Raw N	%	Survey-Weighted N	Raw N	%	Survey-Weighted N	Raw N	p-value
**Overall**	--	9,361,219	7,712	24.74	2,315,614	1,907	75.26	7,045,605	5,805	
**Any Mental Health Treatment**	51.36	4,780,688	4,029	57.25	1,313,299	1,105	49.43	3,467,389	2,924	0.012
**Counseling from a Healthcare Professional[a,b]**	42.07	3,309,210	2,791	49.25	953,293	785	39.73	2,355,918	2,006	0.001
**Prescriptions[a]**	25.96	2,066,338	1,777	36.54	711,801	606	22.54	1,354,537	1,171	<0.001
**Professional Counseling or Prescriptions[a]**	45.11	3,598,199	3,043	54.92	1,072,734	885	41.93	2,525,465	2,158	<0.001
**Alternative Help[a,c]**	3.22	253,051	201	3.50	67,673	64	3.13	185,378	137	0.618
**Inpatient**	6.35	592,812	508	11.57	266,120	230	4.65	326,692	278	<0.001
**Virtual**	31.34	2,925,614	2,410	38.89	895,243	731	28.87	2,030,371	1,679	<0.001
**Through School Resources**	19.13	1,780,046	1,555	19.00	435,511	376	19.18	1,344,535	1,179	0.930
**Specialty Facility**	34.81	3,240,297	2,711	40.33	925,238	761	33.00	2,315,058	1,950	0.002

[a] Questions asked to adolescents experiencing negative feelings
[b] Professional counseling from a healthcare professional includes medical professionals such as physicians, psychiatrists, nurses, and occupational therapists, and includes mental health professionals, including psychologists, psychotherapists, social workers, counselors.
[c] Alternative help includes counseling from a religious leader or advisor and providers of alternative medicine such as herbalists, acupuncturists, or message therapists
The denominator includes respondents who had experienced a mental health diagnosis (a major depressive episode or suicidal ideation) in the past year.

© 2025 Lee D et al. *JAMA Health Forum.*

eTable 4: Differences in socioeconomic status and treatment receipt by age for adolescents with substance use disorder, 2021-2022								
Category	Variable	Age Categories						P-Value
		12-17			18-20			
		%	Survey-Weighted N	Raw N	%	Survey-Weighted N	Raw N	
Overall	Total with SUD		2,360,211	1,981		2,721,549	2,101	
	Total with SUD and MH		1,196,322	1,030		1,119,293	877	
Mental Health Treatments*	Any Mental Health Service	62.75%	743,211	654	51.37%	570,087	451	0.034
	Received Counseling from a Healthcare Professional	49.84%	511,666	447	48.58%	441,627	338	0.796
	Received Prescriptions	37.14%	385,802	333	35.84%	325,999	273	0.804
	Received Professional Counseling or Prescriptions	53.94%	562,881	500	56.06%	509,852	385	0.673
	Alternative Help	3.86%	39,675	35	3.08%	27,999	29	0.678
	Inpatient	14.28%	169,850	151	8.67%	96,271	79	0.020
	Telehealth	38.64%	460,659	400	39.15%	434,584	331	0.912
	Through School Resources	29.89%	353,450	306	7.40%	82,061	70	<0.001
	Specialty Facility	47.15%	558,389	474	33.06%	366,850	287	0.003
Socioeconomic Variables	Insured	94.57%	2,232,100	1,920	90.06%	2,450,913	1,862	0.047
	Medicaid Insurance	47.01%	1,109,577	974	32.69%	889,680	726	<0.001
	Private Insurance	49.08%	1,158,285	958	55.65%	1,514,431	1,069	0.020
	Other Insurance	4.80%	113,216	128	6.80%	185,006	187	0.066
	Received Government Assistance	33.64%	793,981	699	24.75%	673,644	545	0.001
	Poverty	20.13%	475,220	436	25.95%	705,203	605	0.017
	Arrested	6.42%	149,753	139	10.15%	271,480	215	0.026

© 2025 Lee D et al. *JAMA Health Forum*.

Category	Variable	Age Categories						P-Value
		12-17			18-20			
		%	Survey-Weighted N	Raw N	%	Survey-Weighted N	Raw N	
**Substance Use Treatments**	Received Any SUD Treatment	11.45%	270,231	290	8.03%	218,450	181	0.009
	Received Help for Alcohol	3.10%	73,224	76	2.26%	61,538	64	0.327
	Received Help for Drugs	7.89%	186,258	197	5.00%	136,126	116	0.031
	Inpatient	3.03%	71,508	72	2.16%	58,820	46	0.295
	Outpatient	3.55%	83,889	95	3.21%	87,435	69	0.718
	Telehealth	3.78%	89,123	95	2.79%	75,835	56	0.262
	Specialty Facility	5.76%	136,051	142	3.95%	107,536	97	0.056

* The denominator for the mental health treatment variables are adolescents with SUD who had a mental health diagnosis in the past year

© 2025 Lee D et al. *JAMA Health Forum*.

eTable 5: Treatment Receipt by Need and Adolescent Characteristics														
	SUD[a] Treatment (%) for those with SUDs							MH[b] Treatment (%) for those with MH Diagnoses[c]						
	Row = 1 (%)			Row = 0 (%)				Row = 1 (%)			Row = 0 (%)			
**Variables**	**%**	**Survey-Weighted N**	**Raw N**	**%**	**Survey-Weighted N**	**Raw N**	**P-value**	**%**	**Survey-Weighted N**	**Raw N**	**%**	**Survey-Weighted N**	**Raw N**	**P-value**
**Demographic**														
**Female**	10.90%	296,013	278	8.15%	192,668	193	0.118	55.53%	3,383,715	2,855	43.45%	1,396,973	1,174	<0.001
**Hispanic**	8.68%	119,172	122	9.96%	369,509	349	0.643	45.96%	1,073,349	822	53.17%	3,707,338	3,207	<0.001
**White**	10.12%	271,557	231	9.06%	217,123	240		57.91%	2,847,289	2,402	44.03%	1,933,398	1,627	
**Black**	9.02%	51,734	57	9.69%	436,947	414		36.29%	387,036	338	53.31%	4,393,652	3,691	
**Asian**	14.80%	22,292	11	9.46%	466,389	460		47.39%	229,826	127	51.58%	4,550,861	3,902	
**Other**	7.97%	23,925	50	9.72%	464,756	421		48.23%	243,187	340	51.54%	4,537,500	3,689	
**Aged 12-13**	15.66%	49,676	54	9.21%	439,005	417	0.017	50.43%	721,771	670	51.53%	4,058,917	3,359	0.002
**Aged 14-15**	12.66%	95,264	90	9.09%	393,417	381		57.61%	1,251,987	1,049	49.46%	3,528,700	2,980	
**Aged 16-17**	9.71%	125,291	146	9.58%	363,390	325		51.95%	1,278,175	1,148	51.15%	3,502,513	2,881	
**Aged 18-20**	8.03%	218,450	181	11.45%	270,231	290		47.13%	1,528,755	1,162	53.62%	3,251,933	2,867	
**Socioeconomic**														
**Insured**	9.89%	463,288	447	6.37%	25,393	24	0.260	52.29%	4,583,720	3,886	36.35%	196,967	143	0.001
**Medicaid Insurance**	11.41%	228,112	247	8.45%	260,569	224	0.041	51.30%	1,800,886	1,528	51.40%	2,979,801	2,501	0.963
**Private Insurance**	8.63%	230,692	204	10.71%	257,989	267	0.150	53.84%	2,796,512	2,366	48.23%	1,984,176	1,663	0.002
**Other Insurance**	18.21%	54,293	45	9.08%	434,388	426	0.010	46.47%	299,492	291	51.72%	4,481,196	3,738	0.144

© 2025 Lee D et al. *JAMA Health Forum*.

	SUD[a] Treatment (%) for those with SUDs							MH[b] Treatment (%) for those with MH Diagnoses[c]						
	Row = 1 (%)			Row = 0 (%)				Row = 1 (%)			Row = 0 (%)			
**Variables**	**%**	**Survey-Weighted N**	**Raw N**	**%**	**Survey-Weighted N**	**Raw N**	**P-value**	**%**	**Survey-Weighted N**	**Raw N**	**%**	**Survey-Weighted N**	**Raw N**	**P-value**
**Received Government Welfare**	12.56%	184,345	192	8.42%	304,336	279	0.039	52.46%	1,280,102	1,094	50.97%	3,500,586	2,935	0.515
**Poverty**	10.32%	121,785	120	9.41%	366,818	350	0.602	48.68%	894,560	809	52.03%	3,882,494	3,214	0.188
**Arrested**	18.91%	79,667	83	8.81%	404,064	381	<0.001	58.04%	153,974	159	51.22%	4,619,650	3,858	0.234
**Mental Health**														
**MDE**	11.97%	218,393	224	8.23%	259,938	228	0.003	53.17%	3,968,214	3,343	43.39%	768,906	642	0.001
**MDE Role Impairment**	12.78%	182,244	191	8.25%	284,127	248	0.001	58.33%	3,178,402	2,686	40.90%	1,481,553	1,239	<0.001
**Suicidal Ideation**	10.86%	168,154	199	8.36%	251,616	205	0.083	54.78%	2,881,902	2,442	45.41%	1,187,619	977	0.002
**Any Mental Health Diagnosis**	11.34%	262,539	275	8.25%	221,519	188	0.039	X						
**Substance Use**														
**Substance Use Disorder**	X							57.25%	1,313,299	1,105	49.43%	3,467,389	2,924	0.012
**Any Opioid Use**	15.12%	222,066	228	7.38%	266,615	243	<0.001	56.69%	1,073,602	941	50.00%	3,707,085	3,088	0.021
**Opioid Misuse**	25.31%	123,988	113	7.94%	364,693	358	<0.001	62.39%	263,535	231	50.83%	4,517,153	3,798	0.008
**Treatment Rates Overall**	9.62%	488,681	471	90.38%	4,593,078	3,611		51.36%	4,780,687	4,029	48.64%	4,527,663	3,627	

© 2025 Lee D et al. *JAMA Health Forum*.

[a] SUD = Substance use disorder
[b] MH = Mental health
[c] Mental health diagnoses include experiencing a major depressive episode or suicidal ideation in the past year.

© 2025 Lee D et al. *JAMA Health Forum*.

III) Sensitivity Analysis: Modified Poisson Regressions

In this section, we conduct multivariable modified Poisson regressions and compare the resulting p-values with that from our bivariate analysis involving chi-square statistical tests.

A) Sensitivity for Table 1:

eTable 6: Combined Modified Poisson Regression for Substance Use Disorder and Demographic Characteristics			
	Chi-square	Modified Poisson Regression	
Variables	P-values	Coefficient	P-values
Demographic			
Female	<0.001	0.037	<0.001
Hispanic	<0.001	0.022	0.743
Black		-0.266	0.002
Asian		-0.657	<0.001
Other		0.190	0.036
Aged 14-15	<0.001	0.722	<0.001
Aged 16-17		1.223	<0.001
Aged 18-20		1.585	<0.001
Socioeconomic			
Medicaid Insurance	0.976	0.034	0.674
Private Insurance	0.595	0.037	0.660
Other Insurance	0.375	-0.175	0.131

© 2025 Lee D et al. *JAMA Health Forum*.

	Chi-square	Modified Poisson Regression	
**Variables**	**P-values**	**Coefficient**	**P-values**
**Received Government Assistance**	0.104	0.136	0.052
**Poverty**	0.589	-0.046	0.460
**Arrested**	<0.001	0.890	<0.001
**Mental Health**			
**Any Mental Health Diagnosis**	<0.001	0.817	<0.001
**Substance Use**			
**Any Opioid Use**	<0.001	0.608	<0.001

The outcome in this table is the presence of a substance use disorder. The baseline groups are male for gender, White for race, ages 12-13 for ages, and uninsured for health insurance. Our statistical tests with the multivariable modified Poisson regression have similar results as the bivariate tests.

© 2025 Lee D et al. *JAMA Health Forum*.

B) Sensitivity Analysis for Table 3

eTable 7: Modified Poisson Regression of Treatment Rates by Age for Adolescents with Substance Use Disorder						
		Chi-square	Modified Poisson Regression			
			Overall Treatment		Granular Treatment Variables	
**Category**	**Variable**	**P-Value**	**Coefficient**	**P-values**	**Coefficient**	**P-values**
**Mental Health Treatments**	Any Mental Health Service	0.034	-0.230	<0.001		
	Received Counseling from a Healthcare Professional	0.796			0.156	0.081
	Received Prescriptions	0.804			0.303	<0.001
	Alternative Help	0.673			0.268	0.144
	Inpatient	0.020			0.016	0.931
	Telehealth	0.912			0.265	0.001
	Through School Resources	<0.001			-1.421	<0.001
	Specialty Facility	0.003			-0.291	0.001
**Substance Use Treatments**	Received Any SUD Treatment	0.009	0.151	0.083		
	Received Help for Alcohol	0.327			-0.238	0.428
	Received Help for Drugs	0.031			-0.477	0.049
	Inpatient	0.295			-0.387	0.208
	Outpatient	0.718			0.340	0.096
	Telehealth	0.262			0.455	0.090
	Specialty Facility	0.056			0.095	0.761

© 2025 Lee D et al. *JAMA Health Forum*.

		Chi-square	Modified Poisson Regression			
			Overall Treatment		Granular Treatment Variables	
**Category**	**Variable**	**P-Value**	**Coefficient**	**P-values**	**Coefficient**	**P-values**
**Socioeconomic Variables**	Medicaid Insurance	<0.001	-0.473	<0.001	-0.601	<0.001
	Private Insurance	0.020	-0.233	<0.001	-0.248	0.025
	Other Insurance	0.066	0.011	0.823	-0.073	0.608
	Received Government Assistance	0.001	-0.181	<0.001	-0.019	0.837
	Poverty	0.017	0.350	<0.001	0.288	<0.001
	Arrested	0.026	0.528	<0.001	0.508	<0.001

The outcome in this table the indicator for being an older adolescent (aged 18-20) vs. a younger adolescent (aged 12-17) for adolescents with substance use disorders. In general, our statistical tests with the multivariable Poisson regression have similar results as the bivariate tests, with the exception for the new significance of receiving a prescription for negative feelings, telehealth for mental health care and the non-significance of any substance use disorder treatment and the receipt of government assistance.

© 2025 Lee D et al. *JAMA Health Forum*.

C) Sensitivity Analysis for Table 4

eTable 8: SUD Combined Modified Poisson Regression for Treatment Receipt by Need and Adolescent Characteristics						
	With Substance Use Disorders			With Mental Health Diagnoses		
	Chi-square	Modified Poisson Regression		Chi-square	Modified Poisson Regression	
**Variables**	**P-values**	**Coefficient**	**P-values**	**P-values**	**Coefficient**	**P-values**
**Demographic**						
**Female**	0.118	0.247	0.221	<0.001	0.238	<0.001
**Hispanic**	0.643	-0.163	0.465	<0.001	-0.232	<0.001
**Black**		-0.327	0.123		-0.480	<0.001
**Asian**		0.234	0.557		-0.202	0.111
**Other**		-0.426	0.076		-0.181	0.019
**Aged 14-15**	0.017	-0.207	0.459	0.002	0.115	0.068
**Aged 16-17**		-0.375	0.188		0.018	0.746
**Aged 18-20**		-0.572	0.012		-0.073	0.249
**Socioeconomic**						
**Medicaid Insurance**	0.041	0.314	0.121	0.963	0.115	0.074
**Private Insurance**	0.150	0.159	0.467	0.002	0.167	0.004
**Other Insurance**	0.010	0.862	<0.001	0.144	0.007	0.932
**Received Government Assistance**	0.039	0.266	0.222	0.515	0.091	0.046
**Poverty**	0.602	-0.060	0.757	0.188	-0.024	0.605
**Arrested**	<0.001	0.774	<0.001	0.234	0.122	0.194

© 2025 Lee D et al. *JAMA Health Forum*.

	With Substance Use Disorders			With Mental Health Diagnoses		
	Chi-square	Modified Poisson Regression		Chi-square	Modified Poisson Regression	
Variables	P-values	Coefficient	P-values	P-values	Coefficient	P-values
**Mental Health**						
Any Mental Health Diagnosis	0.039	0.194	0.250			
**Substance Use**						
Any Opioid Use	<0.001	0.594	<0.001	0.021	0.115	0.027

The outcomes in this table are the receipt of substance use treatment for adolescents with substance use disorders and mental health treatment for those with mental health diagnoses. In general, our statistical tests with the multivariable Poisson regression have similar results as the bivariate tests, with the exception for the new significance of government assistance for mental health care and the non-significance of Medicaid and mental health diagnoses for substance use treatment and age for mental health treatment.

© 2025 Lee D et al. *JAMA Health Forum*.

eReferences

1. Center for Behavioral Health Statistics and Quality. 2021 National Survey on Drug Use and Health Public Use File Codebook [Internet]. 2022 [cited 2024 Apr 18];Available from: https://www.datafiles.samhsa.gov/sites/default/files/field-uploads-protected/studies/NSDUH-2021/NSDUH-2021-datasets/NSDUH-2021-DS0001/NSDUH-2021-DS0001-info/NSDUH-2021-DS0001-info-codebook.pdf
2. Center for Behavioral Health Statistics and Quality. 2022 National Survey on Drug Use and Health Public Use File Codebook [Internet]. 2023 [cited 2024 Apr 18];Available from: https://www.datafiles.samhsa.gov/sites/default/files/field-uploads-protected/studies/NSDUH-2022/NSDUH-2022-datasets/NSDUH-2022-DS0001/NSDUH-2022-DS0001-info/NSDUH-2022-DS0001-info-codebook.pdf

© 2025 Lee D et al. *JAMA Health Forum*.
