# Supplementary material for: Adolescent Treatment Landscape of Depression, Suicidality, and Substance Use Disorder in the US
Source: JAMA Health Forum. 2025 Aug 29;6(8):e252647. doi: 10.1001/jamahealthforum.2025.2647 (PMC12397884; doi:10.1001/jamahealthforum.2025.2647)
Supplement: Supplement 2. — Data sharing statement [file jamahealthforum-e252647-s002.pdf]

Data Sharing Statement

Lee. Adolescent Treatment Landscape of Depression, Suicidality, and Substance Use Disorder in the US. *JAMA Health Forum*. Published August 29, 2025.
doi:10.1001/jamahealthforum.2025.2647

Data
Data available: No

Additional Information
Explanation for why data not available: The National Survey on Drug Use and Health (NSDUH) is a publicly available dataset provided by the Substance Abuse and Mental Health Services Administration (SAMHSA).
